# Clinical and imaging characteristics of cardiac magnetic resonance presenting with myocardial infarction with non-obstructive coronary arteries in China

**DOI:** 10.1186/s13019-022-02072-6

**Published:** 2022-12-22

**Authors:** Xinxiang Zhao, Daobing Zeng, Liping He, Wenjing Sun

**Affiliations:** 1 Department of Radiology, The Second Affiliated Hospital of Kunming Medical University, No. 374, Dian-mian Avenue, Wuhua District, Kunming, 650101 China; 2 Department of PET Center, Taihe Hospital, Hubei University of Medicine, Shiyan, China; 3 Department of Epidemiology and Biostatistics, School of Public Health, Kunming Medical University, Kunming, China; 4 Department of Cardiology, Henan Provincial People’s Hospital, No. 7, Weiwu Road, Jinshui District, Zhengzhou, China

**Keywords:** Myocardial infarction, Coronary artery disease, Magnetic resonance imaging

## Abstract

**Background:**

The characteristics are still unclear due to lack of systematic research on patients with myocardial infarction non-obstructive coronary arteries (MINOCA) in China. This study aimed to explore the clinical and imaging features of MINOCA patients.

**Methods:**

The patients who were diagnosed as suspected MI were studied. Cardiac magnetic resonance (CMR) was performed after coronary angiography or coronary computed tomographic angiography examination within one week. Myocardial infarction (MI) was determined by late gadolinium enhancement CMR.The patients with MI were divided into MINOCA and MICAD group according to whether the degree of coronary stenosis was greater than 50%. Cardiac function and imaging characteristics between the two groups were analyzed.

**Results:**

21 patients with MINOCA and 30 patients with myocardial infarction with obstructive coronary artery disease (MICAD) were analyzed. MINOCA patients were younger, and the electrocardiogram was commonly featured by non-ST-elevation. The parameters of left ventricular function were significantly different between the two groups including left ventricular ejection fraction, stroke volume, cardiac output, myocardial mass, and peak ejection rate (*P* < 0.05). Besides, MINOCA patients had smaller area of MI, less score of transmural extent, fewer involved segments. Furthermore, the transmural extent of MI in MINOCA patients was mainly grade I, that is, most of them were subendocardial MI, which was significantly negatively correlated with the amount of first-pass perfusion.

**Conclusions:**

The clinical characteristics combined with imaging features of CMR may be effective to evaluate the cardiac function in order to make clinical decision for MINOCA patients in China.

## Background

Acute myocardial infarction (AMI) is a common critical disease with high morbidity and mortality, and almost 6% patients with AMI have MINOCA [1]. As we known, MINOCA patients should meet the diagnostic criteria for AMI, with coronary stenosis of less than 50% [2]. Previous research has reported that the segments of myocardial infarction (MI) without significant stenosis are common as well [3]. However, the clinical and imaging characteristics are still unclear due to lack of systematic research on patients with MINOCA in China.

European Society of Cardiology (ESC) proposes the concept of MINOCA, and points out that the loss of myocardial viability determined by imaging is an important basis for the diagnosis of MINOCA [4]. Recently, multiple methods of detection for myocardial activity are available, such as positron emission tomography/computed tomography (PET/CT), single-photon emission computed tomography (SPECT) and cardiac magnetic resonance (CMR) [5–7]. However, PET-CT and SPECT have ionizing radiation damage and have the features of low spatial resolution. The major advantages of CMR consist of high resolution tissue and no radiation damage. In addition, different aspects can be assessed accurately including wall motion abnormalities, T2 weighted detection of edema, microvascular obstruction, and myocardial viability [8, 9]. Currently, misdiagnosis often occurs in patients with MINOCA, which leads to the aggravation of the disease and the occurrence of adverse events. Thus, it is urgent to detect the imaging characteristics of patients with MINOCA using CMR in order to provide the valuable information for clinical decision.

The aim of this study was to explore the clinical and imaging features of patients with MINOCA. Our study demonstrated that most of patients with MINOCA had non-ST segment elevation and better myocardial viability than patients with MICAD. In addition, segments with MI were mainly located in anterior wall, anteroseptal wall, and inferoseptal wall, which were dominated by the LAD coronary artery. Furthermore, transmural extent of MI in MINOCA patients was mainly grade I, that is, most of them were subendocardial MI, which was significantly negatively correlated with the amount of first-pass perfusion.

## Methods

### Patients

**Fig. 1:**
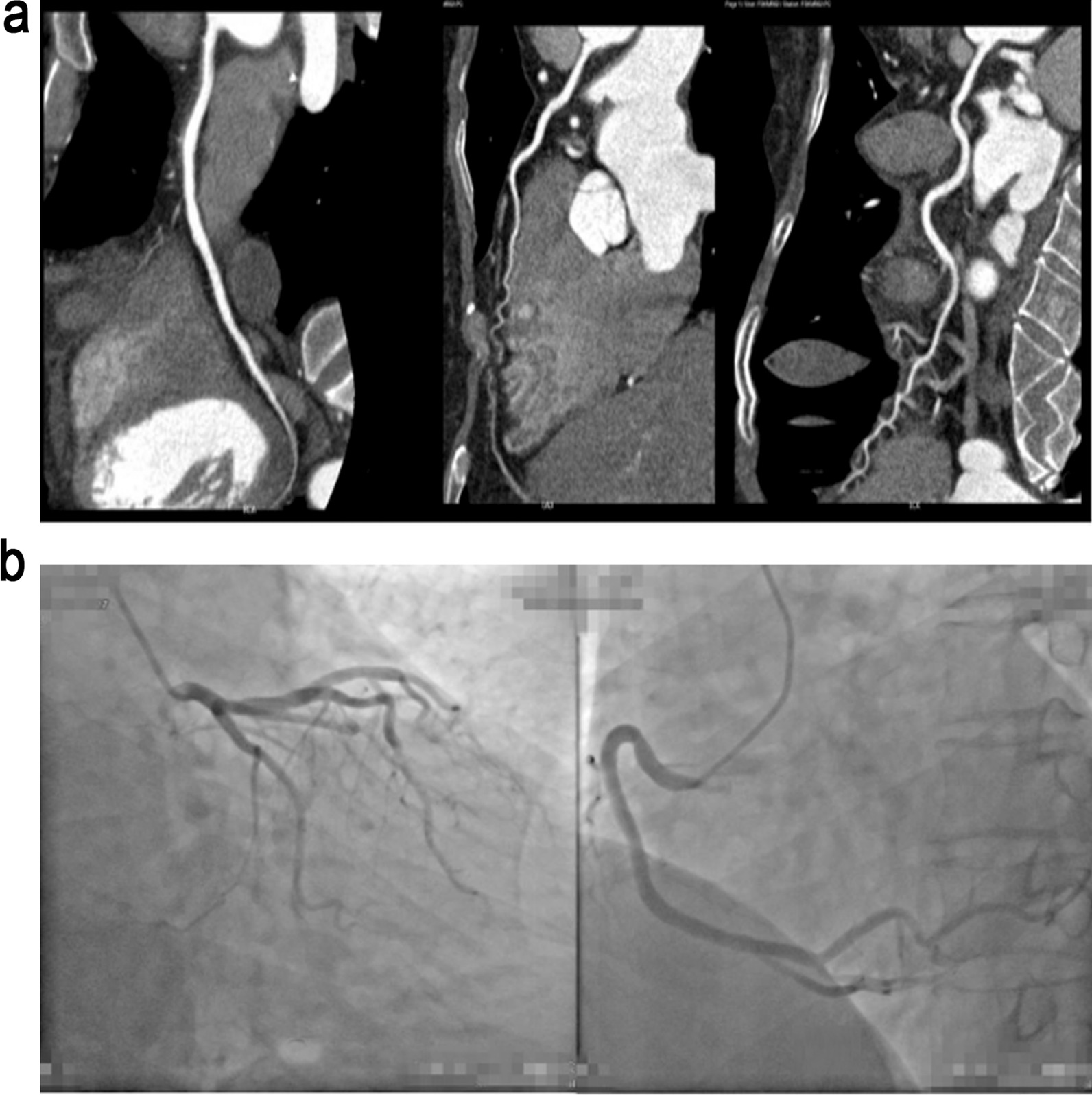
Representative images of coronary computed tomographic angiography (CTA) and coronary angiography (CAG) in two cases. **a** CTA images indicated that no obvious stenosis was observed in coronary arteries (RCA, LAD, and LCX) in a 48-year-old female patient with MINOCA. **b** CAG images indicated that mild stenosis (less than 50%) was observed in RCA coronary artery in a 74-year-old male patient with MINOCA

A total of 21 patients with MINOCA were screened out of patients who were diagnosed as suspected AMI but without significant coronary artery obstruction on CAG or coronary computed tomographic angiography (CTA) from January 2013 to February 2018 in the our hospital (Fig. 1), which was defined as MINOCA group. The included patients met diagnostic criteria of MINOCA published by ESC [4]. Exclusion criteria were patients who (1) suffered with a previous history of MI; (2) suffered with percutaneous coronary intervention (PCI) or coronary artery bypass grafting (CABG); (3) had history of heart diseases and congestive heart failure; (4) had glomerular filtration rate below 30 ml/min/1.73 m^2^; (5) had contraindications using CMR or lack of rationality according to the judgement by researchers.

**Fig. 2:**
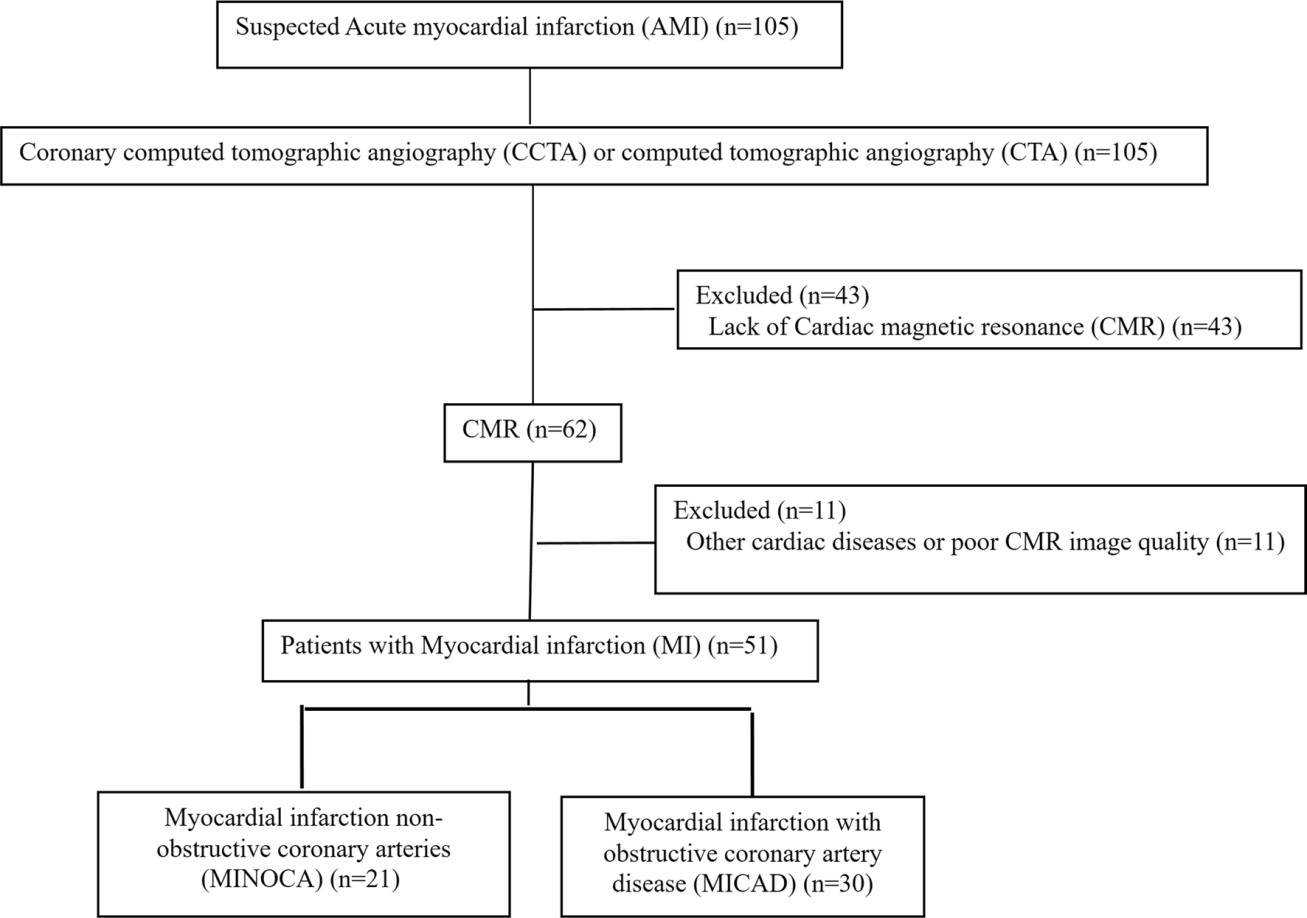
Flowchart of this study

A total of 30 patients with MICAD were selected from patients who were hospitalized and clinically diagnosed as MI with coronary artery stenosis greater than 50% from January 2013 to February 2018 in our hospital, which was defined as MICAD group. Inclusion criteria of patients with MICAD were: (1) CCTA or CAG was completed during the study period, and CMR was completed within one week. (2) Stenosis is greater than 50% in any one of the coronary arteries. (3) LGE-CMR showed the presence of MI, and the infarcted segment was located in the blood supply area with coronary stenosis greater than 50%. The reason for selecting 30 patients was to match the number of patients in the MINOCA group as much as possible. MINOCA group finally included 21 patients, and MICAD selected the top 30 patients who met the criteria during the same period. All patients underwent CMR examination and determination of myocardial infarct segments were using late gadolinium-enhanced (LGE)-CMR. The MICAD group included acute ST-segment elevation myocardial infarction (STEMI) [10] and non ST-segment elevation myocardial infarction (NSTEMI). Exclusion criteria were patients who (1) suffered with other cardiac diseases such as dilated cardiomyopathy, hypertrophic cardiomyopathy, and myocarditis; (2) suffered from severe arrhythmia; (3) had contraindications to magnetic resonance examination or are allergic to contrast agents; (4) refused to join the study. The flowchart were shown in Fig. 2.

### Definition of clinical indicators

Hypertension was defined as office systolic blood pressure values ≥ 140 mmHg and/or diastolic blood pressure values ≥ 90 mmHg in the absence of antihypertensive medication or current antihypertensive treatment. Diabetes mellitus was defined as having a clear history of diabetes or diabetes treatment. Diagnosis of dyslipidemia is based on “Chinese guidelines on prevention and treatment of dyslipidemia in adults (2016 revised edition)” [11]. In brief, dyslipidemia was defined as total cholesterol (TC) ≥ 5.2 mmol/l (200.0 mg/dl), low-density lipoprotein cholesterol (LDL-C) ≥ 3.4 mmol/l (130.0 mg/dl), high-density lipoprotein cholesterol (HDL-C) < 1.0 mmol/l (40 mg/dl) and triglycerides (TG) ≥ 1.7 mmol/l (150 mg/dl). The diagnosis of obesity was established based on body mass index (BMI) > 30 kg/m^2^.

The study was approved by the Institutional Ethics Committee of our hospital and allowed exemption from informed consent. All procedures were performed in accordance with the World Medical Association’s Declaration of Helsinki.

### Clinical data collection

Clinical characteristics including patient’s gender, age, admission symptoms, statistics of risk factors including hypertension, diabetes, dyslipidemia, obesity (BMI > 30 kg/m^2^), smoking history, examination results of 12-lead electrocardiogram and myocardial injury marker were collected.

### CMR examination

CMR imaging was performed on a 3.0 T clinical whole-body scanner (Achieva 3.0 T TX, Philips Healthcare, Best, Netherlands) including the following steps. Images were captured by balanced turbo field echo (B-TFE) sequence (single shot, slice thickness 10.0 mm, flip angle 50°, field of view 311 × 340 mm) in the orientations of 2-chamber-long axis, 4-chamber and short-axis views covering the entirety of the left ventricular from base to apex. During repeated breath-holds in expiration, cine imaging was performed using cine sequence (TR 39.76 ms, TE 1.22 ms, slice thickness 8 mm, field of view 276 mm × 340 mm).

Gadolinium-diethylenetriamine pentaacetic acid (Gd-DTPA) was administered intravenously by power injection with a dosage of 0.08 mmol/kg body weight and a flow rate of 5 ml/s. Following 8–15 min delay, the images were obtained approximately using breath-hold phase-sensitive inversion recovery prepared turbo field echo (PSIR-TFE-BH) sequence. Parameters of the sequence contained long axis slice thickness was 5 mm, short-axis slice thickness was 8 mm, layer spacing was 0 mm, TR was 5.4 ms, TE was 1.0 ms, field of view was 380 × 280 mm, matrix was 220 × 192, flip angle was 25° and TI was 280–380 ms.

### CMR image analysis

**Fig. 3:**
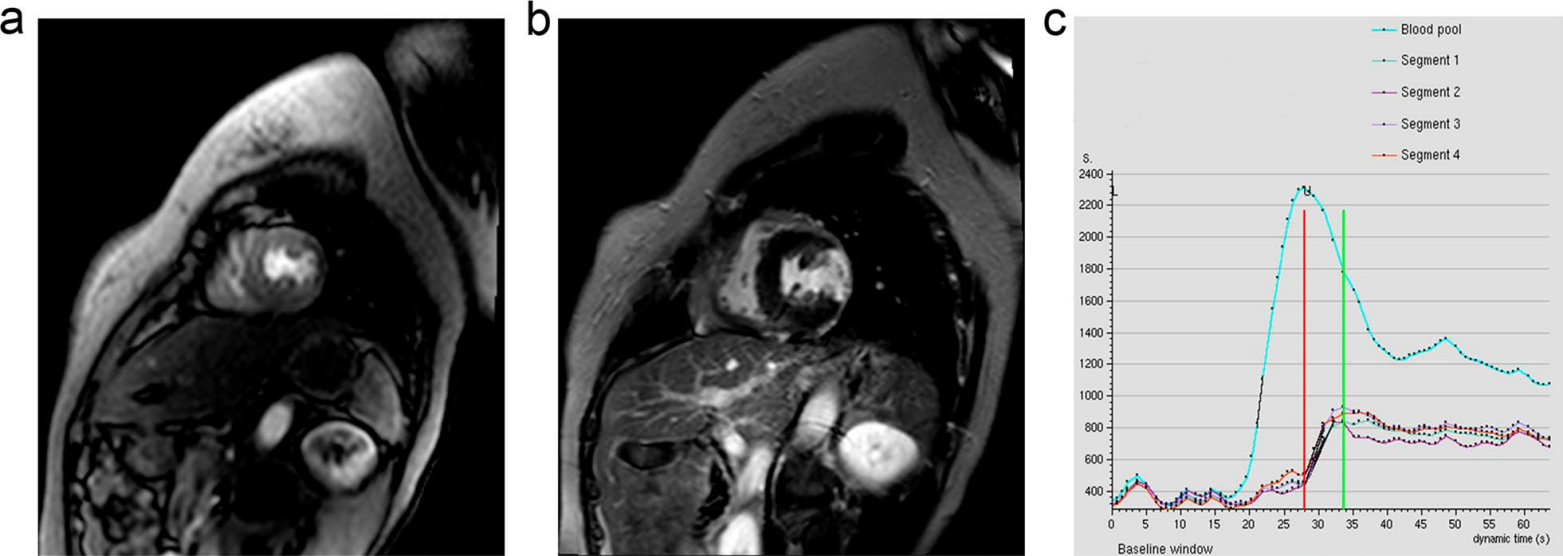
Representative images of cardiac magnetic resonance (CMR) in the 48-year-old female patient with MINOCA. **a** Myocardial first-pass perfusion magnetic resonance imaging indicated apical perfusion defect of left ventricular. **b** Late gadolinium enhancement (LGE) imaging indicated that lateral wall of apical cap (16th segment) occurred the transmural myocardial infarction, and it was supplied by LCX coronary artery. Besides, the transmural extent was grade IV, and area of myocardial infarction was 17.8%. **c** Signal intensity curves indicated that signal intensity of 16th segment decreased at the apical cap of left ventricular apex

All images were interpreted by the consensus of two experienced observers who were blinded to electrocardiographic (ECG), laboratory, and angiographic results. CMR was performed after coronary angiography (CAG) or coronary computed tomographic angiography (CCTA) examination within one week. This study focused on patients in the acute phase within 1 month. On the images obtained by CMR, the division of left ventricular segmentation and the corresponding blood supply is based on the American Heart Association 17-segment model [12]. Transmural extent of myocardial infarct was classified as 5 grades: grade 0: normal, scored 0; grade I: ≤ 25%, scored 1; grade II: 26%–50%, scored 2; grade III: 51%–75%, scored 3; and grade IV ≥ 75%, scored 4 (Fig. 3).

### Statistical analysis

Statistical analysis was performed using SPSS version 17.0 (SPSS, Chicago, IL, USA). The categorical data were analyzed using χ^2^ test and expressed as rate or percentage. The quantitative data were presented expressed as means ± standard deviation (SD). The differences between various groups were analyzed by a one-way ANOVA followed by the LSD-*t* test, and the data of the two groups was assessed using the Student’s* t* test. Kendall correlation analysis was used to assess statistical correlation between myocardial perfusion and transmural extent. *P* < 0.05 was considered statistically significant.

## Results

### Baseline characteristics between the groups

21 patients with MINOCA and 30 patients with MICAD were included in our study. Baseline characteristics of the patients were listed in Table 1. There were no significant differences in BMI value, gender and coronary risk factor (history of hypertension, diabetes, obesity and coronary heart disease) between the two groups (*P* > 0.05).

**Table 1:** Baseline characteristics between the two groups

	MINOCA group	MICAD group	*P* value
Age (years)	47.8 ± 10.98	59.6 ± 11.22	0.001
BMI (kg/m^2^)	23.83 ± 3.27	24.07 ± 3.85	0.820
Male	17 (81.0%)	24 (80.0%)	0.612
Smoking history	5 (23.8%)	22 (73.3%)	0.001
Hypertension	9 (42.9%)	12 (40.0%)	0.838
Diabetes	1 (4.8%)	4 (13.3%)	0.391
Obesity	1 (4.8%)	2 (6.7%)	0.634
Dyslipidemia	6 (28.6%)	21 (70.0%)	0.004
Hyperhomocysteine	11 (52.4%)	24 (80.0%)	0.036
Family history of Coronary heart disease	1 (4.8%)	2 (6.7%)	0.776
Electrocardiogram (ST segment elevation/non-ST segment elevation)	7/14	21/9	0.010
CTnT(ng/ml)	0.56 ± 0.43	1.11 ± 0.57	0.025

*BMI* body mass index, *MINOCA* myocardial infarction with nonobstructive coronary arteries, *MICAD* myocardial infarction with obstructive coronary artery disease

However, patients of MINOCA group were younger than that of MICAD group, and the numbers of smokers in the MINOCA group were fewer than that in the MICAD group (*P* < 0.05). The electrocardiogram was commonly featured by non-ST segment elevation in MINOCA patients, while ST segment elevation in MICAD patients. The cTnT level in the MINOCA group was significantly lower than that in the MICAD group (*P* < 0.05). In addition, the rates of dyslipidemia and hyperhomocysteine in the MINOCA group were lower than MICAD group (*P* < 0.05).

### Cardiac structural and functional parameters between the groups

The results of CMR were shown in Table 2, the rates of wall motion abnormalities and ventricular aneurysm in the MINOCA group were lower than those in the MICAD group (*P* < 0.05). In terms of left ventricular function parameters, left ventricular ejection fraction (LVEF), stroke volume (SV), cardiac output (CO), myocardial mass (MM), and peak ejection rate (PER) in the MINOCA group were significantly higher than those in the MICAD group (*P* < 0.05). However, the peak filling rate (PFR) in the MINOCA group was notably lower than that in the MICAD group (*P* < 0.05). Additionally, left ventricular end-diastolic volume (LVEDV), left ventricular end-systolic volume (LVESV), and cardiac output index (CI) were not significantly different between the two groups (*P* > 0.05).

**Table 2:** The structural and functional parameters between the two groups

	MINOCA group	MICAD group	*P* value
Ventricular aneurysm (presence/absence)	2/19	12/18	0.025
Abnormal regional wall movement (presence/absence)	5/16	16/14	0.046
LVEF (%)	51.85 ± 11.38	39.90 ± 13.34	0.002
LVEDV (ml)	107.1 ± 41.42	91.47 ± 36.24	0.227
LVESV (ml)	50.62 ± 18.95	56.70 ± 28.98	0.159
SV (ml)	56.46 ± 28.82	34.75 ± 13.63	0.001
CO (l)	3.72 ± 1.09	2.30 ± 0.75	0.001
CI (l/min)	2.46 ± 0.68	2.06 ± 0.73	0.053
MM (g)	109.46 ± 37.78	63.38 ± 20.20	0.001
PFR (ml/s)	2.06 ± 0.55	2.51 ± 0.36	0.001
PER (ml/s)	2.83 ± 0.41	2.52 ± 0.47	0.018

*LVEF* left ventricular ejection fraction, *LVEDV* left ventricular end-diastolic volume, *LVESV* left ventricular end-systolic volume, *SV* stroke volume, *CO* cardiac output, *MM* myocardial mass, *CI* cardiac output index, *PFR*, peak filling rate, *PER* peak ejection rate, *MINOCA* myocardial infarction with nonobstructive coronary arteries, *MICAD* myocardial infarction with obstructive coronary artery disease

### Comparison of LGE-CMR between two groups

As shown in Table 3, the area of MI, the scores of transmural extent and number of involved segments in the MINOCA group were less than those in the MICAD group (*P* < 0.05). However, there was no obvious difference in the sites of MI (*P* > 0.05).

**Table 3:** Comparison of LGE-CMR between two groups

	MINOCA group	MICAD group	*P* value
The area of myocardial infarction (%)	12.35 ± 8.94	19.88 ± 9.44	0.006
Involved segments	4.00 ± 2.05	5.60 ± 2.47	0.019
Scores of transmural extent	7.90 ± 5.19	14.50 ± 7.66	0.001
Transmural myocardial infarction (presence/absence)	7/14	20/10	0.025
Sites of myocardial infarction (anterior/non-anterior)	15/6	21/9	0.912

*MINOCA* myocardial infarction with nonobstructive coronary arteries, *MICAD* myocardial infarction with obstructive coronary artery disease

### The location of MI segments in patients with MINOCA

**Fig. 4:**
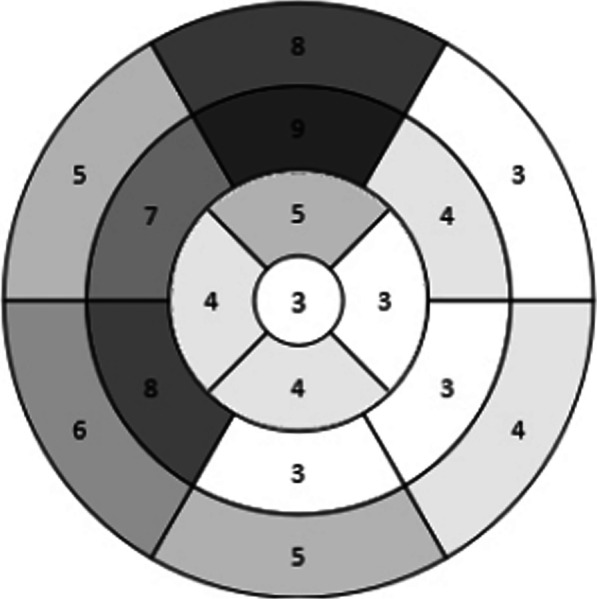
The distribution of myocardial infarction segments in patients with MINOCA

Furthermore, a total of 357 myocardial segments in 21 MINOCA patients were analyzed and found that 84 segments caused MI. The distribution of segments of MI showed 3 segments in the apex, 16 segments in the apical cap, 34 segments in the mid-cavity, and 31 segments in the base (Fig. 4). Among them, 52 segments of MI mainly located in anterior wall, anteroseptal wall, and inferoseptal wall, which accounted for 61.9%. Besides, the incidences of MINOCA in the apex, apical cap, mid-cavity, and base were 14.28%, 19.05%, 26.98%, and 24.60%, respectively. Furthermore, the relationship between left ventricular segmentation and coronary blood supply showed that segments of MI were mostly dominated by the left anterior descending (LAD), indicating the myocardium dominated by LAD had a high probability of infarction.

### Comparison of myocardial perfusion with transmural extent of MI in patients with MINOCA

The transmural extent in 81 segments of MI (apex segments not included) was shown in Table 4. In brief, 41 segments belonged to grade I, 21 segments belonged to grade II, 10 segments belonged to grade III, 9 segments belonged to grade IV. Those finding suggested that the transmural extent of MI in MINOCA patients was mainly grade I, that is, most of them were subendocardial MI.

**Table 4:** Comparison of myocardial perfusion with transmural extent of myocardial infarction in patients with MINOCA

	Grade 0	Grade I	Grad II	Grade III	Grade IV
Segments with normal perfusion	233	1	1	0	0
Segments with reduced perfusion	22	19	7	2	2
Segments with perfusion defect	0	11	13	8	7
Total	255	41	21	10	9

*MINOCA* myocardial infarction with nonobstructive coronary arteries

Furthermore, we analyzed the first-pass perfusion curves of 336 myocardial segments (apex segments not included) in patients with MINOCA and the results showed that the number of segments with abnormal myocardial were more than those of MI, indicating the range of abnormal myocardial perfusion was greater than that of MI (Table 4). The results of Kendell rank correlation demonstrated that the amount of first-pass perfusion was significantly negatively correlated with the transmural extent of MI (τb = − 0.819, *P* = 0.025).

## Discussion

Our study investigated the clinical and imaging features of CMR presenting with patients with MINOCA. The major findings were (1) MINOCA patients were younger, and the electrocardiogram was commonly featured by non-ST-elevation; (2) Patients with MINOCA had smaller area of MI and score of transmural extent, fewer number of involved segments, and better heart function; (3) Segments of MI were mainly located in anterior wall, anteroseptal wall, and inferoseptal wall, which were mostly dominated by the LAD coronary artery, indicating the myocardium dominated by LAD coronary artery had a high probability of infarction; (4) the transmural extent of MI in MINOCA patients was mainly grade I, that is, most of them were subendocardial MI. These findings of this study may provide valuable information for clinical decision making and identify the cause of MINOCA.

Previous studies have confirmed that age, gender, dyslipidemia, hypertension, smoking, diabetes and impaired glucose tolerance, obesity, and family history are risk factors for coronary heart disease [13–15]. With regards to clinical characteristics of MINOCA patients, previous study [1] has demonstrated that MINCOA patients is more likely to be young and less likely to have hyperlipidemia compared with MICAD patients, whereas the smoking history is not statistically different between MINCOA patients and MICAD patients. On the contrary, Daniel et al. [16] have reported that the numbers of smokers are fewer among MINOCA patients. Our results were consistent with previous study has reported in terms of MINOA correlation with smoking history and age. Furthermore, meta-analysis has found that 1/3 MINOCA patients presented with ST segment elevation MI, and 2/3 MINOCA patients presented with non-ST segment elevation MI [17]. Subsequently study has revealed that pathological feature of non-ST segment elevation MI is focal or subendocardial MI [18]. In addition, Raparelli et al. [19] have reported that cTnT plays an important role on MINOCA patients. In our study, the most common of electrocardiographic features was non-ST segment elevation MI in MINOCA patients, and most of them were subendocardial MI. The value of cTnT in the MINOCA group was lower than that in the MICAD group. However, hyperhomocysteine has proved to be closely related to coronary atherosclerosis [17]. Our study found that MINOCA patients had less hyperhomocysteine than those in MICAD patients, which may be due to the lower extent of coronary atherosclerosis in the MINOCA group.

Cine CMR imaging is widely used to evaluate myocardial structure and function in clinical study including heart structure, and movements of valve and ventricular wall [20]. After scanning, the left ventricular function parameters were obtained by post-processing software. In current study, the rates of wall motion abnormalities and ventricular aneurysm in the MINOCA group were lower than those in the MICAD group. Additionally, left ventricular function parameters in the MINOCA group were superior to those in the MICAD group including LVEF, SV, CO, MM, PER, and PFR, indicating patients with MINOCA had better cardiac function. Considering that the reason is related to the etiology of MINOCA. Leurent et al. [21] have indicated that the etiology of MINOCA patients is complicated, however there is no obvious obstruction during coronary angiography, indicating that the degree of coronary atherosclerosis is mild or there is no sclerosis. It may be related to the recovery of coronary arteries blood supply after the spasm relieved or autolysis of thrombosis, so that degree of myocardial damage is less in the patients with MINCOA compared with patients with MICAD, and cardiac function is relatively better than MICAD.

Previous studies have confirmed that the quality of left ventricular myocardium has important value in risk stratification and prognosis assessment of coronary heart disease [22]. Moreover, Mahnken et al. [23] have indicated that the severity and duration of MI is closely associated with the location, scope and extent. In our study, we found that MINOCA patients had smaller area of MI, less score of transmural extent, and fewer involved segments. Those results may be related to the etiology of MINOCA. Reynolds et al. [24] have revealed that myocardium dominated by coronary plaque rupture showed larger edema area and smaller infarct area using CMR, indicating that coronary blood flow is restored immediately after plaque rupture. In addition, our study showed that segments of MI were mainly located in anterior wall, anteroseptal wall, and inferoseptal wall, which were mostly dominated by the LAD coronary artery. Surprisingly, a similar study has demonstrated that culprit lesions were mainly located in the LAD, and there were no obvious difference between the patients with MINOCA and MICAD [25], which was consistent with no obvious difference in the sites of MI between the two groups in our study. Furthermore, transmural extent of MI in MINOCA patients was mainly grade I, that is, most of them were subendocardial MI, which were accordance with previous study reported [24]. Therefore, those results suggested that patients with MINOCA had better myocardial viability and heart function, and myocardium dominated by LAD had a high probability of infarction.

Previous study has indicated that the area of MI, involved segments, and score of transmural extent are the key factors for ventricular remodeling [26]. Additionally, Lund et al. [27] have reported that the specificity and sensitivity of left ventricular remodeling are 92% and 94% when the MI area is greater than 24%, and the probability of ventricular remodeling increases almost triples when the MI area is increased by 10%. Moreover, transmural extent plays an important impact on the prognosis of patients and transmural MI is prone to ventricular aneurysm and ventricular arrhythmias [28]. Our results showed that transmural extent of MI in MINOCA patients was mainly grade I, and it verified the good cardiac function compared with MICAD patients. Those finding suggested that imaging features of CMR may be effective to evaluate the cardiac function in order to make clinical decision for MINOCA patients in China.

## Limitations

We acknowledged that our study has several limitations. First, sample size is limited, and more patients should be included in future research. Second, the data was from only one medical center, more center studies are needed. In addition, another limitation of our study etiology of MINOCA patients is not explored using intravascular ultrasound (IVUS) due to the high cost of IVUS examination.

## Conclusions

Patients with MINOCA were younger, and had fewer smoking history, lower cTnT level and rates of dyslipidemia and hyperhomocysteine. Additionally, the electrocardiogram of patients with MINOCA was commonly featured by non-ST-elevation. Furthermore, the clinical characteristics combined with imaging features may be effective to evaluate the cardiac function in order to make clinical decision for MINOCA patients in China.

## Data Availability

All data generated or analyzed during this study are included in this published article.

## Abbreviations

MINOCA: Myocardial infarction non-obstructive coronary arteries
MICAD: Myocardial infarction with obstructive coronary artery disease
CMR: Cardiac magnetic resonance
CAG: Coronary angiography
CTA: Computed tomographic angiography
LVEF: Left ventricular ejection fraction
SV: Stroke volume
CO: Cardiac output
MM: Myocardial mass
PER: Peak ejection rate
AMI: Acute myocardial infarction
ESC: European Society of Cardiology
PET/CT: Positron emission tomography/computed tomography
SPECT: Single-photon emission computed tomography
LAD: Left anterior descending
PCI: Percutaneous coronary intervention
CABG: Coronary artery bypass grafting
B-TFE: Balanced turbo field echo
Gd-DTPA: Gadolinium-diethylenetriamine pentaacetic acid
PSIR-TFE-BH: Phase-sensitive inversion recovery prepared turbo field echo
ECG: Electrocardiographic
IVUS: Laboratory, angiographic and intravascular ultrasound
SD: Standard deviation
LVEF: Left ventricular ejection fraction
SV: Stroke volume
CO: Cardiac output
MM: Myocardial mass
PER: Peak ejection rate
PFR: Peak filling rate
LVESV: Left ventricular end-systolic volume
CI: Cardiac output index
LAD: Left anterior descending
